# A Feasibility Assessment of Measuring Acceptance and Adherence to a Plant‐Based (Vegan) Diet in a Cardiology Clinic

**DOI:** 10.1002/clc.70438

**Published:** 2026-08-07

**Authors:** L. Smillie, M. Minehan, C. R. Knight‐Agarwal, Ahmad Farshid

**Affiliations:** ^1^ Discipline of Nutrition and Dietetics University of Canberra Bruce Australia; ^2^ Cardiology National Capital Private Hospital Garran Australia

**Keywords:** cardiovascular disease, diet indices, plant‐based, vegan

## Abstract

**Background:**

Plant‐based vegan diets (PBVDs) improve cardiometabolic risk markers, hence are recommended as an adjunct treatment by some cardiologists. To complete a service evaluation, practical measures of dietary adherence are required, and perceptions of patients need to be considered.

**Methods:**

A single‐arm, mixed‐methods design was used to assess the feasibility of using two diet indices to assess adherence to a PBVD and the acceptability of adopting a PBVD in a cardiac outpatient setting. Diet adherence was measured via absence of proscribed foods (APF) and a plant‐based vegan diet score (PBVD‐S). Acceptability of the PBVD was assessed via individual interviews and a food acceptability questionnaire.

**Results:**

Twenty patients completed the dietary assessment. After 8 weeks, 55% (*n* = 11) were classified as fully adherent to the PBVD. Mean score for the PBVD‐S increased from 20.3 ± 8.7 at baseline to 38.9 ± 5.8 at Week 8. Mean total cholesterol (TC), body mass index (BMI), body mass, and waist circumference significantly decreased (*p* < 0.001) after 8 weeks on the PBVD. The PBVD‐S satisfied the feasibility criteria of alignment with diet education and assessment of adherence. The PBVD was rated as either “moderately” or “extremely” acceptable by 80% of patients.

**Conclusions:**

The PBVD‐S is a feasible option for assessing adherence and diet quality of a PBVD in a cardiology outpatient setting.

## Graphical abstract

A plant‐based vegan diet in cardiac outpatients was feasible and well accepted, with 55% achieving full adherence. Improvements in PBVD score, cholesterol, BMI, weight, and waist circumference were observed over 8 weeks. The PBVD score is a useful tool for assessing adherence and diet quality.

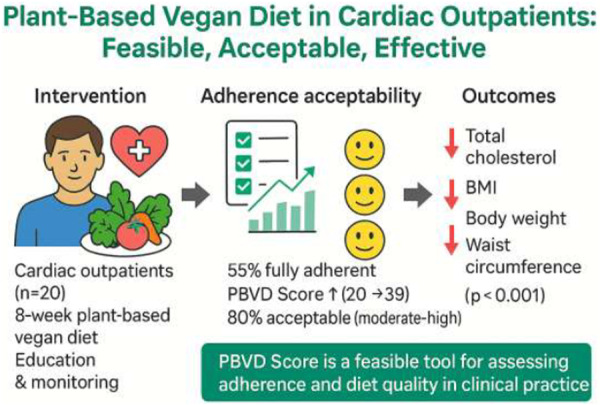

## 1 Introduction

Plant‐based diet (PBD) is an umbrella term referring to dietary patterns that are high in plant foods (fruits, vegetables, nuts, seeds, legumes, and wholegrains) and low or absent in meat and dairy foods [1]. A vegan diet is a type of PBD that completely excludes all foods of animal origin. PBDs contribute to reduced risk of primary and secondary coronary heart disease (CHD) [2]. Meta‐analyses of randomized controlled trials of vegan interventions show improvements in several cardiometabolic risk markers, including body weight and blood lipids [3, 4]. For example, Termannsen et al. found that in comparison with control diets, vegan diets reduced body, body mass index (BMI), glycated hemoglobin, total cholesterol (TC), and low‐density lipoprotein cholesterol in people with overweight or diabetes [3].

Despite clear evidence supporting the benefits of PBDs, advice to adopt a PBD is generally not a focus for management of CHD [5]. The reasons for this are unclear but may relate to a perception that patients are unable or unwilling to significantly alter their eating habits. Potential barriers to clinicians providing dietary advice include a lack of time or insufficient nutritional expertise [6]. Few studies have explored acceptability and adherence to PBDs in cardiology patients [7]. Evaluating a cardiology service that routinely prescribes a PBD will identify opportunities to enhance service delivery and provide a model for other cardiology clinics [8].

Diet acceptability refers to “an individual's judgment of the advantages and disadvantages of a therapeutic diet in relation to palatability, costs, and effects on eating behavior and health—that influence the likelihood of adherence” [9]. Acceptability can be assessed via tools such as the food acceptability questionnaire (FAQ) and/or interviews [10]. Adherence to PBD interventions has been assessed in various ways. Some researchers define adherence as the absence of proscribed foods (APF) [10]. This offers a quick and easy way to assess adherence but offers limited insight into the overall quality of the diet or the nature of dietary change. Diet quality indices (DQI) are predefined scoring systems to evaluate how closely an individual's eating pattern aligns with dietary recommendations [11]. DQIs that accommodate vegan dietary patterns include the plant‐based dietary index (PDI) [12], the Alternate Healthy Eating Index (AHEI‐2010) [13], and the Swiss‐based diet quality score for vegans (DQS‐V) [14]. However, these do not align closely with education centered around food groupings in the Australian Guide to Healthy Eating (AGHE) [15]. In order to be used in a service review, the DQI needs to be quick and easy to analyze, align with nutrition counseling and yield insightful information about adherence and diet quality.

The aims of this study were to assess the (i) feasibility of using two diet indices to assess adherence to a PBVD; (ii) to descriptively examine changes in anthropometric and biochemical measures over 8 weeks in the context of a feasibility study; and (iii) acceptability of adopting a PBVD in a cardiac outpatient setting.

## 2 Subjects and Methods

### 2.1 The Setting

The setting is a private cardiology outpatient practice located in Canberra, Australia. The clinic typically sees 425 new cardiology patients per year. Most patients are encouraged to adopt a PBVD by the cardiologist and referred to an Accredited Practicing Dietitian (APD) for counseling.

### 2.2 Standard Diet Guidance

Patients interested in adopting a PBVD attend a 90 min appointment with an APD with expertise in PBVDs. A nutrition assessment is conducted to facilitate personalized nutrition advice that caters for dietary preferences, secondary health issues, allergies, and intolerances [16]. Dietary education covers how to consume adequate nutrients; how to optimize cardio‐protective nutrients (e.g., soluble fiber and omega‐3 fatty acids); recommendations for main meals and snacks; a suggested meal plan; a recipe booklet and instructions to take a vitamin B12 supplement (500 µg) daily [17]. Patients are advised to avoid or limit meat, fish, poultry, eggs, and dairy foods while increasing plant‐based foods (fruits, vegetables, wholegrains, legumes, nuts and seeds, calcium‐rich foods) in line with vegan choices within the AGHE [15]. Patients are counseled to incorporate small portions of discretionary foods (DF) (high saturated fat, salt, alcohol) to enhance enjoyment and thus sustain behavior change [18]. Weight loss is not discouraged for overweight patients; however, weight loss is not a specific focus of the dietary guidance. Patients are instructed to eat until they are satiated. Table 1 provides an example of diet parameters.

**Table 1:** Example of diet parameters and example meal plan provided as standard practice.

Definition of intervention diet	Example daily PBVD meal plan
No animal products (meat, fish, poultry, eggs, dairy).	Breakfast: Porridge made with 1 cup dry oats + 1 cup calcium fortified soy milk + 1 banana + 2 tbsp ground linseeds
Emphasis on unprocessed plant‐based foods (per day)^a^	
5 serves vegetables	Lunch: Minestrone soup + 2 slices wholegrain
2 serves fruit	bread + 3 dried figs
4 serves wholegrains	Snack: 2 wholegrain crispbread + avocado
2 tablespoons nuts and seeds	Dinner: Tofu stir fry (170 g tofu + 1 cup cooked
2.5 serves legumes	brown rice + 2 cups vegetables)
2.5 serves calcium‐rich foods	+ 1 cup cubed rockmelon
2 serves unsaturated fats	
Limit discretionary/processed foods (high saturated fat, salt, sugar, alcohol)	
10 µg B12 supplement daily	

a Quantities as per AGHE Standard Serves [15].

Patients can call or email the APD for ongoing support and guidance regarding the PBVD. They also attend a 30 min appointment with the dietitian at Week 4 for review and additional guidance. During these sessions, information such as meal ideas, optimizing nutritional adequacy, and meal preparation is provided.

### 2.3 Study Design

This study used an 8‐week, single‐arm design to complete a feasibility assessment to inform a future service evaluation. As a single‐arm feasibility study, the study was not designed or powered to evaluate the effectiveness of the PBVD on clinical outcomes, and any observed changes in anthropometric or biochemical measures are reported descriptively only.

Ethics approval was granted by the University of Canberra Human Research Ethics Committee (13655). The study assessed current practice, hence did not require registration as a clinical trial. All patients provided written informed consent to share their existing data and participate in dietary assessment and interviews.

### 2.4 Participants

All new patients of the cardiology clinic within a 6‐month period were invited to participate in the study. Interested patients voluntarily contacted the lead researcher (L.S.) who explained the study requirements and confirmed eligibility. Exclusion criteria were: (i) patients who had not been advised to adopt a PBVD by the cardiologist, and (ii) patients who already followed a PBVD. We did not exclude patients on any medication, including statins. The clinical care of all patients was overseen by a registered cardiologist (A.F.).

### 2.5 Sample Size Justification

There are varying approaches to determining sample sizes for studies that assess feasibility [19]. As our primary aim was to understand the feasibility of using different dietary indices to assess dietary adherence, we aimed to recruit 10% of new patients for the study duration (April 1, 2024 and September 27, 2024), giving a target sample of 22 participants. Similar studies have recruited 9–32 participants [20, 21]. We aimed for 80% retention and completion.

### 2.6 Dietary Adherence

A 24‐hr dietary recall (24hrDR) and diet interview were administered by L.S. at baseline, Week 4, and Week 8. The 24hrDR collected detailed information on all foods and beverages consumed on the day prior to interview, from midnight to midnight [22]. Adherence to the PBVD was examined using two indices.

### 2.7 Adherence Defined as the APF

Adherence to the PVBD was defined as the absence of any proscribed foods (meat, poultry, fish, dairy, or eggs) reported via 24hrDRs and diet interviews. This was based on the method suggested by Barnard to assess a low‐fat vegan diet [10, 23, 24, 25]. However, we did not include Barnard's thresholds for saturated fat, total fat, and dietary cholesterol as this is not a focus of standard dietary counseling in the clinic. Participants were dichotomized as adherent or nonadherent based on consumption or non‐consumption of animal foods.

### 2.8 Plant‐Based Vegan Diet Score (PBVD‐S)

The PBVD‐S was modeled on the DQS‐V. The DQS‐V is a tool for scoring the diet quality of vegan diets according to Swiss dietary recommendations for vegans [26]. The DQS‐V was selected due to its focus on vegan dietary patterns; however, it was adapted to align with the AGHE and the specific dietary education provided in this clinical setting. This adaptation was necessary to ensure practical applicability, interpretability for clinicians, and consistency with the food group‐based counseling approach used in the clinic [15]. Eight food groups were categorized into two dietary components: (1) adequacy and quality (vegetables; fruits; wholegrains; legumes, nuts and seeds; non‐dairy calcium‐rich foods and unsaturated fats); (2) moderation (meat, chicken, fish, eggs and dairy and DF). DF and drinks are not a necessary part of a healthy diet and are high in saturated fat, added sugars, salt, and/or alcohol [15]. Foods were classified as DF according to the Australian Bureau of Statistics discretionary flag list and criteria in the Australian Health Survey user guide [27]. Each food group was assigned a score of 0, 2.5, 5, or 10 based on the respective recommendations, resulting in a possible PBVD‐S of between 0 and 50. A higher score represents higher adherence to the PBVD. Dietary intake was classified into high (≥ 40), moderate (30–39), low (20–29), or very low (≤ 20) quality based on PBVD‐S (S1). Score thresholds were pragmatically defined to reflect gradations in adherence and diet quality based on the distribution of scores and alignment with recommended intake patterns across food groups. These thresholds were intended to support interpretation within a clinical context rather than represent validated cut‐points. The PBVD‐S represents an adapted tool and has not undergone formal validation. The feasibility of using the APF and PBVD‐S were assessed against five criteria (speed; ease of use; alignment with dietary education; capacity to assess adherence; usefulness to assess the relationship between adherence and outcome measures) using a 5‐point Likert scale where responses were ranked from 1 (*most positive*) to 5 (*most negative*). A lower score indicates better feasibility.

### 2.9 Clinical Measures

Body mass, height, BMI, and weight were measured by LS at baseline and Week 8 as per International Society for Advancement of Kinanthropometry (ISAK) methods [28]. Total serum cholesterol (TC) prior to commencement of the PBVD and after 8 weeks were obtained from patient records. Information on lipid‐lowering medication use (e.g., statins) at baseline was extracted from patient records. Changes in medication type or dosage during the 8‐week period were not systematically recorded. Medication use was therefore not included as a covariate in statistical analyses.

### 2.10 Acceptability and Patient Perspectives

A FAQ [23] was administered at Week 8. Patients answered 10 questions relating to the foods they had been eating on the PBVD such as “how well do you like the foods you have been eating” and “how much effort does it take you to stay on this diet?” using a seven‐point Likert scale. The FAQ items have previously shown adequate test−retest reliability [24] and can be found in the Supporting Information Materials S2.

Individual qualitative interviews were conducted post‐intervention, either face‐to‐face (*n* = 15) or via the online platform Zoom (*n* = 5) by L.S. Mean interview duration was 14.7 ± 4.6 min. Responses to 13 open‐ended questions (Supporting Information Materials data) covering themes such as dietary acceptability and challenges were audio recorded. The recorded interviews were transcribed prior to analysis.

### 2.11 Analysis

Data for AFP, PVBD‐S, servings of food groups, body mass, BMI, waist circumference, and TC are reported as counts and percentages. The distribution of data was assessed using visual inspection of histograms to evaluate approximate normality. Where normal distributions were observed, data were provided as mean and standard deviation. Otherwise, the median and interquartile range are shown. Given the small sample size, formal testing of sphericity was not undertaken. Differences in PBDV‐S, servings of food groups, body mass, BMI, waist circumference, and TC between baseline, Week 4, and Week 8 were analyzed using repeated measures ANOVA. Given the number of outcomes assessed, the potential for type I error was considered. Bonferroni post hoc tests were used to make comparisons between the three time points, and the adjusted *p* values for the comparisons were reported. Significance was set as *p* < 0.05. Qualitative interviews were analyzed using Braun and Clarke's approach to thematic analysis [29]. L.S. coded data and assigned themes. Analysis was interrogated via discussion with M.M. and C.R.K.‐A.

## 3 Results

### 3.1 Participant Baseline Characteristics

Baseline characteristics of participants are summarized in Table 2.

**Table 2:** Baseline characteristics of 21 patients attempting a PBVD.

Characteristics (*N* = 21)	*n* (%)
Age, mean (SD), years	64.3 (8.7)
Female (*n* = 13)	61.7 (8.7)
Male (*n* = 8)	68.5 (7.2)
Smoking status	
Smoker	
Ex‐smoker	6 (28.6)
Never smoked	15 (71.4)
Education	
University or technical college	15 (71.4)
Year 12 or equivalent	5 (23.8)
Some high school	1 (4.8)
Household income	
$25 000−$50 000	
$50 000−$100 000	6 (28.6)
$100 000−$200 000	8 (38.1)
> $200 000	3 (14.3)
Prefer not to say	4 (20)
How many people live in your household?	
One (1)	2 (9.5)
Two (2)	13 (61.9)
Three‐four (3−4)	5 (23.8)
> Four (4)	1 (4.8)
Who does most of the food shopping?	
Participant	10 (47.6)
Shared	8 (38.1)
Someone else	3 (14.3)
Who does most of the food cooking?	
Participant	10 (47.6)
Shared	7 (33.3)
Someone else	4 (20)
I feel confident about being able to cook from basic ingredients	
Strongly disagree	1 (4.8)
Somewhat agree	6 (28.6)
Strongly agree	14 (66.7)
I feel confident about preparing and cooking new foods and recipes.	
Somewhat disagree	2 (9.5)
Somewhat agree	7 (33.3)
Strongly agree	12 (57.1)
How would you rate the adequacy of your kitchen and kitchen appliances to prepare healthy food?	
Extremely inadequate	2 (9.5)
Somewhat inadequate	2 (9.5)
Somewhat adequate	6 (28.6)
Extremely adequate	11 (52.4)

A total of 28 patients expressed interest in the study. After initial screening and explanation of the study protocol, seven patients declined to participate, one due to competing dietary demands and others due to inconvenience. Thus, a total of 21 patients were enrolled in the study. One patient dropped out at Week 3 due to a significant personal event representing 95.2% retention (target 80%). Twenty patients completed the 8‐week intervention, including attendance at all education sessions and pre and post‐intervention assessments, representing 95.2% attendance and completion of surveys (target 80%). Anthropometric data were available for 18 participants at baseline and week 8. TC was available for 17 participants at baseline and Week 8. At baseline, 48% (*n* = 10) of participants were taking lipid‐lowering medication.

### 3.2 Adherence, Diet Indices and Diet Quality

All patients consumed omnivorous diets at baseline, hence adherence was 0% based on APF. At Weeks 4 and 8, 11 (55%) patients were fully adherent to the PBVD based on APF. At baseline, only one participant scored 40 or above for the PBVD‐S. The number of participants scoring 40 or above increased at Week 4 and then again at Week 8. Only one participant scored below 30 at Week 8. At Week 8, the mean PBVD‐S was similar for males (38.9 ± 5.8) and females (39.1 ± 4.6). A repeated measures ANOVA was performed to compare baseline and follow‐up adherence and diet measures of patients (Table 3). The mean PBVD‐S increased significantly from baseline to Week 4 and remained stable at Week 8. Intake of vegetables, fruit, wholegrains, and legumes increased from baseline to Week 8 with statistically significant increases for vegetables (*p* < 0.001) and legumes (*p* < 0.001). Legumes increased significantly from baseline to Week 4 (*p* = 0.001), and vegetables increased significantly from Weeks 4 to 8 (*p* < 0.001). Mean intake of meat and dairy serves (*p* < 0.001), and DF (*p* < 0.05) decreased significantly from Weeks 0 to 4 and continued to decrease from Weeks 4 to 8.

**Table 3:** Repeated measure ANOVA analysis of baseline and follow‐up adherence and diet measures of patients.

Parameter	Week 0	Week 4	Week 8	*F*	*ƞ* ^2^
Adherence (PBVD‐S), mean (SD)	20.3 (8.7)	37.3 (7.7)	38.9 (5.8)	57*	0.75
Vegetable serves	3.0 (1.2)	3.4 (1.2)	4.6 (1.3)	18.8*	0.50
Fruit serves	1.5 (0.9)	2.0 (0.6)	2.3 (0.7)	5.9**	0.24
Wholegrain serves	3.8 (1.7)	4.8 (1.7)	4.6 (1.8)	2.5	0.11
Legumes, nuts, and seeds serve	0.8 (1.2)	2.5 (1.6)	2.9 (1.2)	21.7*	0.53
Meat and dairy serve	3.6 (1.6)	0.9 (1.5)	0.5 (0.8)	51.4*	0.73
DF serves	1.7 (1.8)	0.8 (0.9)	0.4 (0.8)	12.6*	0.50

*Note:* Values are mean (SD); *p* 0−4, *p* values for changes from Weeks 0 to 4; *p* 4−8, *p* values for changes from Weeks 4 to 8; *p* values for from Weeks 0 to 8; Effect size *ƞ* ^2:^ 0.01 (small effect), 0.06 (medium effect), and 0.14 (large effect).

Abbreviations: DF, discretionary food; PBVD‐S, plant‐based vegan diet score.

*p < 0.001; **p < 0.05.

The PBVD‐S demonstrated greater overall feasibility, scoring lower (more favorable) than the APF across most criteria, particularly in alignment with education, assessment of adherence, and usefulness for analyzing relationships with outcomes. In contrast, the APF performed better only on speed and ease of use, resulting in a higher total score.

### 3.3 Exploratory Changes in Anthropometric and TC Measures

Exploratory analysis indicated that when compared to baseline, mean Week‐8 TC showed a statistically significant within‐person decrease of −1.1 ± 1.0 mmol/L (*p* ≤ 0.001). This represents an 18.9% decrease in TC. Significant reductions were also observed for body mass (−2.3 ± 2.1 kg, *p* ≤ 0.001), waist (−2.0 ± 3.7 kg, *p* = 0.025), and BMI (−0.8 ± 0.7 kg/m^2^, *p* = 0.001) (Table 4).

**Table 4:** Paired samples *t*‐test results for body mass, BMI, waist, and total cholesterol after 8‐week PBVD (*n* = 18).

	Week 0	Week 8	*p* value
Body mass (kg)	78.9 (20.3)	76.6 (20.1)	< 0.001*
BMI (kg/m^2^)	26.7 (5.3)	25.9 (5.3)	< 0.001*
WC (cm)	99.3 (14.5)	97.3 (14.3)	0.025*
TC (mmol/L)	5.3 (1.6)	4.3 (1.1)	< 0.001*

*Note:* Values are mean (SD); *Paired sample *t*‐test, values are mean (SD).

Abbreviations: BMI, body mass index; SD, standard deviation; TC, total cholesterol; WC, waist circumference.

### 3.4 Acceptability

Overall patient satisfaction was high with 80% (*n* = 16) of patients being either “extremely” or “moderately” satisfied with the PBVD; 75% (*n* = 15) finding the appearance of the PBVD foods “extremely” or “moderately” appealing and 90% (*n* = 18) liking the taste of the PBVD foods “a great deal” or “a moderate amount.” Eating out was a challenge with 50% (*n* = 10) of patients reporting that it was either “slightly,” “moderately,” or “extremely” difficult to maintain their PBVD at restaurants. 25% (*n* = 5) found it “slightly” or “moderately” difficult to purchase and prepare foods for the PBVD. Patient responses to the FAQ are outlined in Supporting Information Materials S3.

Post‐intervention interviews (*n* = 20) revealed that most patients found the PBVD acceptable and were overall very positive about their experience with the diet. Patients expressed feeling supported by both the APD and their family and friends. Perceived challenges included difficulty in finding, purchasing, and cooking plant‐based food items, and a dislike for the taste of some foods, such as soy milk and tofu. Motivation to continue the diet was enhanced through real or perceived benefits, such as feeling more energetic or learning of improvements to cardiometabolic risk markers. Indicative quotes for qualitative themes can be found in Supporting Information Materials S4.

## 4 Discussion

This study aimed to assess the feasibility of using two diet indices to measure adherence to a PBVD. Both the APF and PBVD‐S were considered feasible methods of measuring adherence; however, the PBVD‐S offered more useful insight into the implementation of the PBVD. The APF allows for a quick and easy assessment of adherence. This method can be utilized by non‐nutrition experts and could be used by patients to self‐assess adherence. Patients can be dichotomized into adherent and nonadherent for analysis. The main limitation of the APF is that it does not account for the quality of dietary change. A PBVD can be poorly constructed; include high salt and high sugar DF and lacks cardio‐protective food sources such as fruits and vegetables [30]. Furthermore, many plant‐based meat alternatives have a poor nutrient profile, are a source of saturated fat, and are considered to be ultra‐processed as per the NOVA classification [31]. When prescribing a PBD in a cardiology setting, it is thus vital to prioritize healthful PBDs with food sources rich in cardio‐protective nutrients such as fiber, polyunsaturated fatty acids, phytochemicals, and certain vitamins and minerals [32]. A tool that assesses diet quality is recommended for future service evaluation.

The PBVD‐S strongly aligned with the education provided in the cardiology clinic. It allowed for an evaluation of the overall diet quality of a vegan dietary pattern. Patients who successfully increased the amount and quality of plant foods but did not fully abstain from animal‐derived foods could be identified with this method. As could patients who excluded animal‐derived foods but continued to consume inadequate vegetables, fruit, and grains. The focus on quality allows for better assessment of cardioprotective foods, such as fruits and vegetables. The PBVD‐S allows for continuous and categorical analysis which extends the capacity for analysis. Given the time constraints typical in clinical settings, DQIs that are straightforward and quick to administer are generally preferred [33]. Nutrition expertise is required to utilize the PBVD‐S, and it is more time‐consuming than the APF. In practice, engaging a nutrition professional, such as an APD, can streamline the application of the PBVD‐S.

A key finding of this study is the practical utility of the PBVD‐S in a real‐world clinical setting. The PBVD‐S aligned closely with the dietary education provided and allowed for rapid assessment of both adherence and diet quality. This has important implications for clinical practice, where time constraints often limit detailed dietary assessment. The PBVD‐S could be integrated into routine dietetic consultations to monitor patient progress, identify specific areas of dietary improvement (e.g., low vegetable or legume intake), and guide personalized feedback. In contrast to binary adherence measures such as the APF, the PBVD‐S provides a more nuanced understanding of dietary behavior, which may enhance patient engagement and support behavior change.

Many healthcare professionals assert that adhering to a PBD is challenging [34]; however, after 8 weeks, 55% of our patients were fully adherent based on the APF. In our sample of 20 patients, PBVD‐S scores increased significantly from a range of 10−40 at baseline to a range of 25−45 at Week 8. Mean score for the PBVD‐S increased from 20.3 ± 8.7 at baseline to 38.9 ± 5.8 at Week 8 indicating most participants had partially adopted a PBVD with moderate diet quality. Our results indicate that cardiology patients can adhere to a quality PBVD diet. This finding aligns with a 2020 review investigating adherence and retention in seven low‐fat vegan intervention studies in individuals with type 2 diabetes. The researchers found that adherence was either “good” or “high” in most studies [35].

Statistically significant reductions in body mass, BMI, waist circumference, and total serum cholesterol occurred in our participants after 8 weeks of partial to full adherence with a PBVD. These observations are consistent with findings from controlled trials of plant‐based diets [36, 37]; however, given the single‐arm design, no causal inference can be made regarding the effect of the PBVD.

Although weight loss was not a focus of our dietary education, patients achieved a mean body mass loss of 2.3 ± 2.1 kg. The magnitude of mean body mass loss is small, falling within the range of typical body weight variability [38] and is lower than that observed in more structured intervention PBD interventions that prescribe energy restriction [39]. Our patients achieved a reduction of 1.1 ± 1.0 mmol/L in TC, representing an 18.8% decrease, which is encouraging, as population‐level data suggest that each 1% decrease in TC is associated with a 2.5% reduction in CVD [40]. A recent meta‐analysis of 29 randomized controlled trials investigating vegan diets reported a mean TC decrease of 0.34 mmol/L (95% confidence interval, −0.44 to −0.23; *p* = 1 × 10^−9^) [41]. The observed reduction in cholesterol may be consistent with increased intake of plant‐based foods, accompanied by a reduction in the intake of red meat and DFs among our patients, although multiple factors (including medication use) may have contributed.

Our changes in anthropometric and TC need to be interpreted with caution. Our small sample included patients with diverse medical presentations, including some with familial hypercholesterolemia. Approximately half of our patients were taking cholesterol‐lowering statin medication at baseline.

We did not specifically recommend sterol‐enriched products, such as fortified spreads or milks, nor did we monitor their use. Consumption of these products is known to have a cholesterol‐lowering effect [42]. We also acknowledge that not all participants necessarily needed to reduce body mass or cholesterol. Weight reduction is not always positive in cardiology patients and could reflect poor implementation of a PBVD with failure to consume sufficient food and kilojoules in total. Our future service evaluation will benefit from considering other measures of successful patient outcomes. For example, whether individual patient goals are met, as well as measures of well‐being and quality of life.

We were successful in recruiting patients who expressed a strong desire to improve their cardiovascular health through lifestyle means. This likely contributed to high acceptability findings, with 80% (*n* = 16) of our patients being either extremely or moderately satisfied with the PBVD. Despite high acceptability, some participants encountered challenges in locating, purchasing, and preparing plant‐based food items, despite the practical dietetic education provided. Known barriers towards adopting a PBD include taste preference, unsupportive family members, and lack of cooking skills [43].

Several patients reported disliking the taste of some of the study foods, such as soy milk and tofu. However, food choices are highly individual and are influenced by a variety of factors including psychological, socio‐cultural, economic, sensory, and knowledge and skills [44, 45]. Personalizing nutrition advice is one approach that may assist individuals navigate challenges like these when modifying their food choices, particularly when supported by a dietitian [45, 46].

Emerging evidence suggests that plant‐based dietary patterns may influence cardiometabolic health through mechanisms beyond traditional risk factors, including potential effects on oral microbiota and systemic inflammation [47]. While this was not assessed in the present study, it highlights an area for future research linking dietary adherence with broader physiological markers.

### 4.1 Strengths and Limitations

Our study successfully assessed the feasibility of using the APF and PBVD‐S in a cardiology clinic and provided useful insight to inform planning for a future service evaluation. Our findings reinforce that it is possible for motivated cardiology patients to partially or fully implement a PBVD and improve diet quality.

We acknowledge that the single‐arm design is limited. The single‐arm design without a control group limits the ability to draw causal conclusions regarding the effect of the PBVD on anthropometric or biochemical outcomes. Observed changes may reflect regression to the mean, concurrent treatments (e.g., statins), or other unmeasured factors.

We opted to use 24hrDRs to record dietary intake. 24hrDRs provide detailed intake data but are susceptible to recall bias and may not capture day‐to‐day variation in food intake (228). Although recalls were conducted at multiple time points, a single 24 h period may not capture day‐to‐day variation in food consumption, particularly for foods consumed irregularly. This is particularly relevant for the assessment of adherence to a PBVD, as participants may have been misclassified as adherent or nonadherent based on a single day's intake. While the PBVD‐S shows promise as a tool to score the diet quality of a vegan diet, it is an adapted index and has not yet undergone formal validation. While it demonstrated feasibility and practical utility in this setting, further research is required to establish its reliability, validity, and sensitivity to dietary change in diverse populations. While our patients reported overall high satisfaction with a PBVD after 8 weeks, longer follow‐up would provide useful insight into the long‐term sustainability of a PBVD. Future controlled studies are required to evaluate effectiveness. A number of statistical comparisons were conducted across dietary and clinical outcomes, increasing the risk of type I error. Although Bonferroni adjustments were applied to post hoc comparisons, the findings should be interpreted cautiously, particularly given the small sample size and exploratory nature of the analyses.

## 5 Conclusion and Future Directions

Combined, the findings of this study suggest that the PBVD‐S is a useful and feasible index to assess adherence to and diet quality of a PBVD in clinical settings. A PBVD is well accepted by motivated patients in a cardiology outpatient setting.

## Author Contributions

Study concept: L. Smillie. Study design: L. Smillie, C. R. Knight‐Agarwal, M. Minehan, and Ahmad Farshid. Data collection: L. Smillie. Data analysis: L. Smillie. Conducted the data analysis with input, review, and editing from M. Minehan, C. R. Knight‐Agarwal, and Ahmad Farshid. L. Smillie wrote the first draft with contributions from M. Minehan, C. R. Knight‐Agarwal, and Ahmad Farshid. All authors reviewed, edited, and agreed on subsequent drafts. The content in this manuscript has not been published elsewhere.

## Funding

The authors have no proprietary or commercial interest in any materials discussed in this article.

## Ethics Statement

The authors of this manuscript certify that they comply with the ethical guidelines for authorship and publishing in Clinical Cardiology.

## Conflicts of Interest

The authors declare no conflicts of interest.

## Supporting information


Supporting File 1

Supporting File 2

Supporting File 3

Supporting File 4

## Data Availability

The data that support the findings of this study are available from the corresponding author upon reasonable request. The data are not public due to privacy or ethical restrictions.
