# Supplementary material for: A Feasibility Assessment of Measuring Acceptance and Adherence to a Plant‐Based (Vegan) Diet in a Cardiology Clinic
Source: Clin Cardiol. 2026 Aug 7;49(8):e70438. doi: 10.1002/clc.70438 (PMC13449299; doi:10.1002/clc.70438)
Supplement: Supplementary file 1 [file CLC-49-e70438-s001.docx]

**Food Acceptability Questionnaire**

1. How well do you like these foods?
2. How well do you like the taste of these foods?
3. How appealing or unappealing do you find the appearance of these foods?
4. How boring are these foods?
5. How easy or difficult has it been for you to prepare these foods?
6. How easy or difficult has it been for you to purchase these foods?
7. How easy or difficult has it been for you to maintain your current diet at restaurants?
8. How much effort does it take for you to stay on this diet?
9. How satisfied or dissatisfied do you feel after eating a meal on this diet?
10. Overall, how satisfied or dissatisfied are you with this diet?
