# Supplementary material for: A Feasibility Assessment of Measuring Acceptance and Adherence to a Plant‐Based (Vegan) Diet in a Cardiology Clinic
Source: Clin Cardiol. 2026 Aug 7;49(8):e70438. doi: 10.1002/clc.70438 (PMC13449299; doi:10.1002/clc.70438)
Supplement: Supplementary file 2 [file CLC-49-e70438-s002.docx]

**S3: Food acceptability questionnaire responses from 20 patients who attempted a PBVD for 8 weeks (n=20)**
