# Supplementary material for: A Feasibility Assessment of Measuring Acceptance and Adherence to a Plant‐Based (Vegan) Diet in a Cardiology Clinic
Source: Clin Cardiol. 2026 Aug 7;49(8):e70438. doi: 10.1002/clc.70438 (PMC13449299; doi:10.1002/clc.70438)
Supplement: Supplementary file 3 [file CLC-49-e70438-s003.docx]

**S1: PBVD-S - food groups and serve sizes used for scoring**

|  | Food Group | Recommendation | Serve size | Scoring Criteria | Scoring |
| --- | --- | --- | --- | --- | --- |
| Adequacy and quality | 1. Vegetables | 5 servings | 75g^a^ | <1 serving  1-< 5 serving  ≥ 5 serving | 0  2.5  5 |
|  | 2. Fruits | 2 servings | 150g | <1 serving  1 - < 2 servings  ≥ 2 servings | 0  2.5  5 |
|  | 3. Wholegrains | 4 servings | 500kJ | < 1 serving  1 - < 4 servings  ≥ 4 servings | 0  2.5  5 |
|  | 4. Legumes, nuts and seeds ^b^ | 2.5 servings | 500-600kJ | <1 serving  1 - < 2.5 servings  ≥ 2.5 servings | 0  2.5  5 |
|  | 5. Vegan milk & yoghurt alternatives^c^ | 2.5 servings | 500-600kJ | < 1 serving  1 - <2.5 servings  ≥ 2.5 servings | 0  2.5  5 |
|  | 6. Unsaturated fats and oils^d^ | 2 serves | 250kJ | < 1 serving  1 - < 2 servings  ≥ 2 servings | 0  2.5  5 |
| Moderation | 7. Meat and dairy^e^ | 0 servings | 500-600kJ meat and/or dairy | ≤ 1 serving  >1, <2.5 servings  ≥ 2.5 servings | 10  5  0 |
|  | 8. Discretionary foods^f^ | Max. 1 serving | 600kJ | ≤ 1 serving  >1 - <2.5 servings  ≥ 2.5 servings | 10  5  0 |
|  | **Total Score** |  |  |  |  |

^a^One serving of a food item was defined according to the AGHE standard serves (27).

^b^Includes legumes, nuts, seeds and minimally processed meat alternatives such as tofu and Textured Vegetable Protein

^c^Includes calcium fortified plant-foods such as plant-milks and yoghurts (minimum 100mg calcium per 100ml)

^d^Includes unsaturated oils, spreads and nut butters

^e^Includes combined meat, poultry, fish, eggs, dairy milk, dairy yoghurt, dairy cheese

^f^As per AGHE definition
