# Supplementary material for: A Feasibility Assessment of Measuring Acceptance and Adherence to a Plant‐Based (Vegan) Diet in a Cardiology Clinic
Source: Clin Cardiol. 2026 Aug 7;49(8):e70438. doi: 10.1002/clc.70438 (PMC13449299; doi:10.1002/clc.70438)
Supplement: Supplementary file 4 [file CLC-49-e70438-s004.docx]

**S4: Supporting quotes from post-intervention interviews with patients attempting a PBVD intervention (n=20)**

|  | Exemplar quotes |
| --- | --- |
| Expectations of a PBVD | *“I went into this trial with a bit of trepidation, you know about going on to a fully plant-based diet. But I found the experience quite uplifting” (P17)*  *“(I thought) it was going to be a little bit harder” (P2)* |
| Support from health professionals | *“Having someone, like yourself, checking in; I'd love to have someone that I saw even once every two months. That just gave (me) all sorts of ideas, like you did with the tofu and different cooking things and different nutritional things; that would be spot on” (P4)*  *“I've been knowing that you're (dietitian) going to be there at the end of it, so I'm going to try and do my best” (P11)* |
| Support from others | *“It was easier than I expected, and I think maybe in part because I did have lots of support from my family” (P9)* |
| Mixed reactions to new foods | *“I actually prefer the soy coffee to regular milk” (P19)*  *“The food we've been eating is tasty. And easy to prepare generally” (P12)*  *“We only tried firm tofu and didn't really find it as a good substitute for meat or fish” (P2)* |
| Reinforced motivation due to real or perceived benefits | *“When those blood tests came in (reduced lipids), I just went –‘Oh my God’ - I can't put my body back into that state, so I think for me, wherever I can have a vegan option, I'll take it” (P13)*  *“I'll definitely continue. I really enjoy it. I like the way it makes me feel” (P4)* |
| Challenges | *“What has been difficult has been finding the right stuff. Not all supermarkets and shops have got what you want” (P15)*  *“It only took a few days for me to become acutely aware of how much I was going to miss butter” (P6)*  *“I found the cooking part was sometimes difficult with not knowing how to prepare some of the (foods)” (P6)* |
| What next | *“I don't know how rigidly I would stick to it after the trial, but I'm pretty sure I will be more plant-based” (P19)*  *“I don't think I'll go back to eating meat……..* *but I do think I will go back to cheese” (P2)* |
